# Alveolar dysregulation of host response in pneumonia and ARDS: implications for immune modulation and infection

**DOI:** 10.1016/j.aicoj.2026.100085

**Published:** 2026-05-21

**Authors:** L.S. Boers, L. Maessen, J. Wauters, E.D. Morrell, A. Sarma, A. Conway Morris, L.D.J. Bos

**Affiliations:** a Department of Intensive Care Medicine, Amsterdam UMC, Location University of Amsterdam, Meibergdreef 9, Amsterdam, the Netherlands; b Department of Microbiology, Immunology and Transplantation, KU Leuven, Leuven, Belgium; c Division of Pulmonary, Critical Care, and Sleep Medicine, University of Washington, Seattle, WA, United States of America; d Division of Pulmonary, Critical Care, Allergy and Sleep Medicine, University of California, San Francisco, CA, United States of America; e Division of Perioperative, Acute, Critical Care and Emergency Medicine, Department of Medicine, and John V. Farman Intensive Care Unit, Addenbrooke’s Hospital, University of Cambridge, Cambridge, United Kingdom

**Keywords:** ARDS, Pneumonia, Alveolar host response, Immune dysregulation, Alveolar injury

## Abstract

**Background:**

Respiratory failure due to pneumonia and acute respiratory distress syndrome (ARDS) remains a major cause of morbidity and mortality in the intensive care unit. The alveolar compartment plays a central role in both pathogen clearance and tissue injury, yet its biology is poorly captured by systemic measurements. Growing evidence shows that dysregulation of alveolar host responses drives disease progression, shapes susceptibility to secondary infections, and influences recovery.

**Main body:**

Community-, hospital-, and ventilator-associated pneumonia differ in microbial drivers and host responses, but all share a pattern of localized inflammation. In most cases, pathogens are rapidly controlled following antimicrobial treatment, while alveolar inflammation persists. This persistence likely reflects self-reinforcing cycles between epithelial injury, neutrophil activity, and monocyte–macrophage dysfunction. Mechanical ventilation further disrupts local defenses, promoting microbial overgrowth and ventilator-associated pneumonia.

In ARDS, diffuse alveolar damage initiates an early influx of neutrophils and monocyte-derived macrophages. Although peripheral blood has been used to identify systemic inflammatory subphenotypes, molecular signatures in the alveolar compartment often differ and may provide complementary biological information. Patients may diverge into distinct alveolar immune trajectories, ranging from sustained alveolar hyperinflammation to immune exhaustion. These divergent trajectories influence downstream repair, determining whether patients achieve epithelial recovery or develop fibrotic remodeling.

Alveolar immune dysregulation also creates a permissive niche for viral and fungal pathogens. Pulmonary reactivation of herpes simplex virus or cytomegalovirus and fungal infections with Aspergillus frequently occur in critically ill patients and may reflect impaired local host defense. These processes are associated with prolonged mechanical ventilation and illustrate how impaired alveolar defenses may sustain injury and propagate complications.

**Conclusion:**

Pneumonia and ARDS share common pathways of alveolar immune dysregulation that are not adequately captured by systemic profiling alone. Integrating systemic and alveolar immune assessment, including their concordance and discordance, may improve patient stratification, facilitate identification of treatable traits, and support the development of more personalized, compartment-informed therapeutic strategies, including both immunomodulatory and pathogen-directed interventions. A more refined understanding of compartment-specific host responses will be key to advancing these approaches.

## Background

Despite advances in supportive care, infection-related respiratory failure remains a major cause of morbidity and mortality in the intensive care unit (ICU). Pneumonia and acute respiratory distress syndrome (ARDS) exemplify the dual challenge of balancing pathogen clearance with limitation of inflammation-mediated tissue injury at the alveolar interface. In these syndromes, the lung is not only a target of injury but also a critical site where host immunity is shaped. The local host response frequently eliminates pathogens, yet can become dysregulated in ways that propagate injury or suppresses defenses to other microbes.

Recent translational studies have reframed our understanding of the alveolar compartment as an active immunological niche with unique dynamics. In contrast to systemic circulation, where immune responses can be readily profiled, the alveolar environment remains clinically elusive, accessible only via invasive procedures and rarely sampled longitudinally. Yet growing evidence suggests that immune dysfunction within this compartment plays a central role in the pathophysiology and complications of critical illness.

Technological advances in single-cell profiling, spatial transcriptomics, and functional immunophenotyping are now illuminating the complexity of immune responses in the alveoli during pneumonia and ARDS. These tools are helping to disentangle the interplay between hyperinflammation and immune suppression, and point toward alveolar-specific mechanisms that underpin local tissue injury, as well as the risk of secondary infection.

This review focuses on the dysregulation of alveolar host immunity in pneumonia and ARDS. The objective of this review is to synthesize current insights into alveolar host response dysregulation and define its pathophysiological context and implications, as a basis for future translational approaches to diagnosis and treatment. We first explore immune dysfunction in community- and ventilator-associated pneumonia, followed by a discussion of injury and host response dysregulation in ARDS. We conclude by addressing how alveolar immune failure contributes to infectious complications such as invasive aspergillosis and herpesvirus reactivation, and what emerging translational approaches might offer for diagnosis and treatment.

## Methods

This narrative review is based on a structured literature search in PubMed and Embase conducted between July 2025 and February 2026, focusing on alveolar host response in pneumonia (CAP, HAP and VAP) and ARDS, including immune dysregulation, secondary infections (viral reactivation and aspergillosis), and mechanisms of lung injury and repair. This was supplemented by reference screening and expert selection of relevant studies.

## Community-, hospital- and ventilator-associated pneumonia

**Fig. 1:**
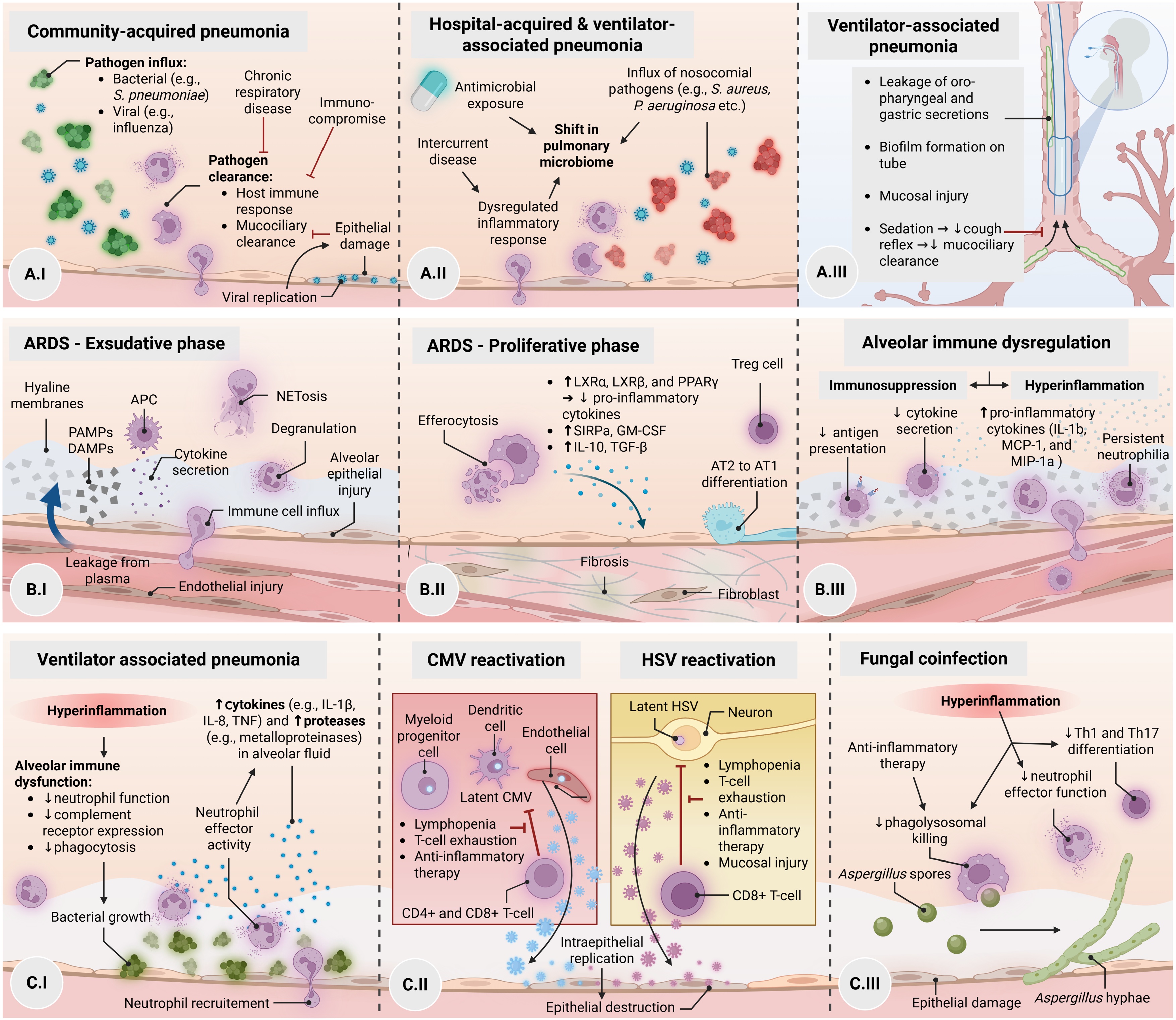
Alveolar immune dysregulation across pneumonia and ARDS. (A) Distinct microbial drivers and host responses characterize community-, hospital-, and ventilator-associated pneumonia (VAP, HAP, and CAP, respectively), with mechanical ventilation further disrupting local defenses. (B) ARDS evolves from an early exudative phase with neutrophil-dominated inflammation toward either resolution with epithelial repair or persistent immune dysregulation and fibrosis. (C) Dysregulated alveolar immunity predisposes to secondary infections, including bacterial VAP, pulmonary HSV/CMV reactivation, and fungal coinfection with *Aspergillus*. Abbreviations: ARDS, acute respiratory distress syndrome; CAP, community-acquired pneumonia; HAP, hospital-acquired pneumonia; VAP, ventilator-associated pneumonia; HSV, herpes simplex virus; CMV, cytomegalovirus.

The human lung is continuously exposed to viruses, bacteria, and fungi via inhalation and aspiration. In health, there is a dynamic equilibrium between microbial influx and clearance by the host defenses that results in a low biomass of varied microbial species along the tracheobronchial tree [1]. Chronic respiratory diseases and changes in immune competence alter the lung microbiome, even in the absence of infection [1]. When the balance is perturbed and microbial influx and growth exceed clearance, this stimulates an immune response intended to kill pathogens, remove damaged tissue, and restore normal lung function, but these processes can also lead to dysregulated and harmful inflammation that stresses the host. The causative microbes and host responses vary greatly by clinical setting, which leads to important differences in the respiratory biology of community-acquired, hospital-acquired, and ventilator-associated pneumonia (CAP, HAP, and VAP, respectively) (Fig. 1A). HAP is defined as pneumonia occurring ≥48 h after hospital admission and not incubating at the time of admission, in patients not receiving invasive mechanical ventilation. VAP refers to pneumonia developing ≥48 h after initiation of invasive mechanical ventilation [2]. Although VAP is considered a subset of HAP, it is typically distinguished due to its specific pathophysiological mechanisms related to mechanical ventilation. Despite these upstream biological differences, downstream alveolar host responses show substantial overlap, particularly characterized by neutrophil-dominated inflammation, epithelial injury, and persistent activation of innate immune pathways.

**CAP** commonly arises from viral or bacterial insults. The most common pathogens are respiratory viruses including rhinovirus and influenza, and *Streptococcus pneumoniae* [3,4]. Whilst CAP may arise in the previously healthy lung, patients with structural lung disease or immunocompromising conditions are higher risk [5]. Viral infections can also injure epithelial barriers and lead to increased susceptibility to secondary bacterial infections [6]. In some cases, severe CAP is characterized by dysregulated immune responses which results in impaired cellular antimicrobial functions, persistent inflammation, and non-resolving lung injury [7]. (Fig. 1A.I)

**HAP** develops in a markedly different milieu than CAP. Hospitalized patients have a shift in microbiota due to factors such as intercurrent illness and antimicrobial exposure [8,9]. Because of this shift, HAP is predominantly caused by a different spectrum of bacterial pathogens than CAP, including *Staphylococcus aureus*, Enterobacterales, and *Pseudomonas aeruginosa* [10], Hospitalized patients can also have impaired peripheral blood and alveolar immune cell functions that precede the development of HAP [[11], [12], [13], [14]], suggesting that the stress of intercurrent disease can increase the risk of developing pneumonia through dysregulated inflammatory responses. (Fig. 1A.II)

**VAP** arises in part from additional disruptions to host defenses introduced by mechanical ventilation, on top of the microbial shifts and dysregulated immunity that contribute to HAP. Endotracheal tubes (ETT) disrupt the typical barrier between the upper airway and lower respiratory tract and oropharyngeal and gastric secretions can leak around the cuff. In addition, sedation and paralysis blunt cough reflexes, and some drugs can impair mucociliary clearance. Bacteria also rapidly form biofilms on ETTs, and these biofilms can become a persistent and difficult to eradicate source of pathogens. Beyond the bacterial pathogens commonly implicated in HAP, the role of non-bacterial organisms in VAP is discussed in more detail later in this review, including fungal pathogens such as *Aspergillus* spp. [15], and an underappreciated incidence of nosocomial viral infections [16] (Fig. 1A.II-III).

With the growing availability of molecular microbiology, we have an increasingly complete picture of causative pathogens in patients with pneumonia [17], although interpretation remains challenging due to issues such as colonization versus infection, contamination, and limited standardization of metagenomic approaches [18]. In animal models, different pathogens have distinct histo-immunological characteristics [19] and exhibit both conserved and pathogen-specific transcriptional responses [20]. In human disease, however, neither clinical nor radiological features can reliably distinguish pathogens [21]. Immunological profiling of the lung remains at an early, experimental stage and shows overlapping patterns across different causal pathogens and apparently non-infectious lung injury [7,22]. Even advanced host–microbe profiling approaches performed in plasma primarily capture systemic immune signatures and provide limited insight into compartment-specific immune processes within the lung, underscoring the need for direct investigation of the alveolar environment [23]. Recent high-dimensional immune profiling of bronchoalveolar lavage fluid suggests that this overlap is primarily driven by shared innate immune activation, particularly neutrophil-dominated responses, whereas pathogen-specific differences appear to be more pronounced at the level of immune activation states rather than cellular composition [24].

The alveolar responses in pneumonia, across CAP, HAP and VAP, characterized by increased cytokines, infiltrating immune cells, and leakage of plasma proteins, reflect compartment-specific yet largely overlapping inflammatory patterns that differ from those observed in the periphery [7,[25], [26], [27]]. Alveolar immune cells mount antimicrobial responses such as phagocytosis, bacterial killing and cytokine production [11,28]. In the presence of appropriate antimicrobial therapy, pathogen burden typically decreases rapidly and is often no longer detectable by conventional microbiological methods, although complete eradication may not always occur [29]. Despite this microbiological clearance, inflammation and lung injury may persist or become self-sustaining, driven by dysregulated host responses rather than ongoing infection, such that inflammation itself contributes to ongoing disease even as the underlying infection resolves, reflecting the frequent overlap between pneumonia and ARDS [30].

What process(es) drive this persistence remains a matter of ongoing investigation, with hypothesized mechanisms including the development of autonomous self-reinforcing cycles and disproportionate responses to low pathogen titers [7,31,32]. Understanding the varied alveolar responses and how these interact with the rest of the body to produce lung injury are likely to be critical to developing effective therapies. The balance of ensuring and enhancing pathogen elimination whilst limiting and ameliorating inflammation and lung injury remains a challenge, and it is likely that individualized therapies will be required. Taken together, infection-driven alveolar inflammation in pneumonia may, in a subset of patients, evolve into or overlap with the dysregulated host responses that characterize ARDS.

## Alveolar injury and host dysregulation in ARDS

Although pneumonia is the most common cause of ARDS, similar patterns of alveolar immune dysregulation can be observed across ARDS from both pulmonary and extrapulmonary causes, including non-infectious etiologies, and may already be present in pneumonia without ARDS. ARDS is characterized by diffuse alveolar damage with epithelial and endothelial injury, resulting in loss of barrier function, proteinaceous edema, and immune cell influx. Although this description is focused on lung-specific injury patterns, prognostic and predictive enrichment approaches based on biomarkers measured from the peripheral blood are increasingly being used to identify biologically distinct subphenotypes of patients with ARDS. As in pneumonia, multiple studies have demonstrated that peripheral blood molecular signatures are often poor surrogates for lower respiratory tract cellular and molecular biology [[33], [34], [35], [36], [37], [38], [39], [40]]. Key studies comparing systemic and alveolar compartment information are summarized in Table 1. More importantly, associations between molecular signatures and clinical outcomes may differ substantially between the lung and blood compartments [33,34,37,38]. This suggests that the alveolar compartment may provide complementary biological information that may help discern subphenotypes or treatable traits in patients with ARDS beyond subphenotypes derived from systemic biomarkers alone.

**Table 1:** Studies comparing systemic (blood) and alveolar compartment information.

Study	Population	Biological sample	Molecular measurement	Key finding (blood vs alveolar)
Conway Morris et al. 2009 [11]	Critically ill patients with suspected VAP	Peripheral blood neutrophils and BALF cells/supernatant	Neutrophil functional assays and complement measurements	Peripheral blood neutrophil dysfunction was mediated predominantly by activated complement/C5a, whereas profound complement-independent impairment occurred in the inflamed alveolar compartment.
Conway Morris et al. 2010 [27]	Critically ill patients with suspected VAP	Serum and BAL fluid	Protein biomarkers	Serum markers showed no discriminatory value for VAP, whereas BALF IL-1β and IL-8 accurately demarcated VAP; the VAP group appeared to have a brisk inflammatory response confined to the lung.
Morrell et al. 2018 [33]	Early ARDS (≤48 h)	Peripheral blood monocytes and BALF-derived alveolar macrophages	Bulk transcriptomics (microarray)	Highly divergent transcriptional profiles; blood does not reflect alveolar gene expression; identical pathways show opposite associations with outcomes.
Bendib et al. 2021 [34]	Moderate-to-severe pneumonia-related ARDS	Blood and BALF	Protein biomarkers and flow cytometry	Alveolar cytokine concentrations exceeded serum levels (most compartmentalized: IL-8); lower BAL fluid-to-serum ratios in shock suggested less lung-compartmentalization and were associated with mortality (IL-1Ra). HLA-DR and PD-1 were higher in the alveolar than blood compartment but not associated with outcomes.
de Brabander et al. 2023 [40]	Mechanically ventilated COVID-19 ARDS	Longitudinal paired plasma and BALF	Protein biomarkers	Biomarkers involved in the innate host response were found in higher concentrations in the alveolar compartment compared with plasma, indicative of a predominant alveolar inflammatory response; longitudinal increases in alveolar biomarker concentrations were associated with mortality, with distinct patterns observed in plasma.
Sathe et al. 2023 [38]	Early ARDS (≤48 h)	Plasma and BALF	Protein biomarkers, LCA	Biomarkers that were clearly different in plasma had minimal differences in BALF; plasma-derived ARDS classes did not consistently reflect early alveolar inflammatory profiles, while BALF identified distinct classes.
Morrell et al. 2024 [37]	ARDS across multiple cohorts	Plasma and BALF/ETA; PBMCs and BALF cells	Soluble checkpoint proteins and mass cytometry	Higher plasma sPD-L1 was associated with mortality, whereas higher lung sPD-L1 was not associated with mortality or was associated with survival. PD-1 expression and intracellular cytokine staining were higher in alveolar than peripheral blood T cells, supporting distinct lung versus blood PD-L1/PD-1 activity.
Xu et al. 2020 [25]	COVID-19 patients (mild and severe)	Plasma and BALF; PBMCs and BALF cells;	Single-cell RNA sequencing; TCR tracking; protein biomarkers	Peripheral monocytes showed an immune-paralyzed/MDSC-like state, whereas BALF monocyte-macrophages produced higher levels of cytokines and chemokines, demonstrating a dichotomy between systemic immune suppression and local pulmonary hyperactivation.
Neyton et al. 2024 [26]	Mechanically ventilated COVID-19 ARDS	Whole blood/PBMCs and tracheal aspirate	Bulk and single-cell RNA sequencing; protein biomarkers	Dexamethasone showed compartment- and cell-specific effects, with discordant gene-expression effects in lung and blood; several interferon-related pathways remained elevated in the respiratory tract but not in blood.

Abbreviations: ARDS, acute respiratory distress syndrome; BALF, bronchoalveolar lavage fluid; C5a, complement component 5a; COVID-19, coronavirus disease 2019; ETA, endotracheal aspirate; HLA-DR, human leukocyte antigen-DR; IL, interleukin; LCA, latent class analysis; MDSC, myeloid-derived suppressor cell; PBMCs, peripheral blood mononuclear cells; PBMs, peripheral blood monocytes; PD-1, programmed cell death protein 1; sPD-L1, soluble programmed death-ligand 1; TCR, T-cell receptor; VAP, ventilator-associated pneumonia.

In ARDS, the alveolar response evolves from an early exudative phase through a proliferative (repair) phase toward either resolution with epithelial recovery or persistent immune dysregulation and fibrosis (Fig. 1B). These trajectories are discussed below and may differ substantially between patients.

### Phases of alveolar response

The initial exudative phase of ARDS is characterized by capillary congestion and intra-alveolar edema, which are key features of diffuse alveolar damage (Fig. 1B.I). Lung epithelial and antigen-presenting cells sense the presence of pathogen-associated molecular patterns (PAMPs) and damage-associated molecular patterns (DAMPs), leading to the release of numerous cytokines and chemokines that help recruit inflammatory cells such as monocytes and neutrophils [[41], [42], [43], [44]]. Neutrophils dominate the alveolar compartment during this early exudative phase and release tissue-damaging mediators including proteases and neutrophil extracellular traps (NETs). In contrast to systemic hyperinflammation, alveolar hyperinflammation can be detected across all phases of ARDS [24,45], raising the question of how local inflammatory responses relate to clinical outcomes.

Mortality in ARDS is rarely driven by respiratory failure alone. Instead, early deaths are predominantly attributable to shock and multi-organ failure, which have consistently been identified as the leading causes of death in ARDS [46,47]. Accordingly, peripheral blood immune signatures such as the hyperinflammatory subphenotype, are strongly associated with early mortality, but not with longer-term outcomes. In contrast, studies employing serial lower respiratory tract sampling have shown that an early alveolar pro-inflammatory response is either not associated with worse outcomes [48] or is associated with improved outcomes compared with less inflammatory molecular signatures [34,49]. This divergence reflects fundamental differences in the biological processes captured by systemic versus alveolar compartments. Together, this suggest that early alveolar inflammation may represent an appropriate local host response to acute lung injury, whereas systemic hyperinflammation primarily reflects disease processes that drive early death.

The subsequent trajectory of ARDS following the initial exudative phase is highly variable and appears to depend on whether early alveolar inflammation appropriately resolves or instead persists in a dysregulated manner. While some patients transition toward a proliferative (or reparative) stage (Fig. 1B.II), others exhibit ongoing inflammatory lung injury or develop alveolar immune suppression characterized by impaired antigen presentation and cytokine production (Fig. 1B.III). Persistence of pro-inflammatory mediators within the alveolar compartment such as interleukin (IL)-1β, C-C motif chemokine ligand 2 (CCL2; formerly MCP-1), and C-C-motif chemokine ligand 3 (CCL3; formerly MIP-1α) [48], as well as sustained inflammatory gene expression within alveolar immune cells [49], have consistently been associated with worse ARDS outcomes [48,49]. Several types of leukocytes have been involved in persistent inflammation, including persistent alveolar neutrophilia and specific neutrophil and monocyte/macrophage subsets. Beyond these cellular processes, ongoing exposure to external factors such as mechanical ventilation–induced stress may contribute to persistence of alveolar injury by sustaining epithelial damage and pro-inflammatory signaling (biotrauma) [50]. Hyperoxemia and hypercapnia may further modulate these processes through effects on epithelial injury, inflammation, and repair, potentially contributing to persistence of lung injury and delayed resolution [51,52]. However, most evidence on these external factors derives from experimental and preclinical models and remains largely limited to the cellular or systemic level, with limited insight into compartment-specific alveolar host responses.

### Heterogeneous trajectories of alveolar immune dysregulation

Following the initial phases of alveolar injury, patients diverge into distinct immune trajectories. Within the alveolar neutrophil compartment, a distinct population of immature CD123^+^ neutrophils that was strongly associated with adverse outcome was recently identified [24]. CD123^+^ alveolar neutrophils displayed an immature phenotype with reduced expression of canonical effector and maturation markers, alongside features consistent with altered activation and prolonged persistence within the alveolar environment. Importantly, the presence of this subset, rather than overall neutrophil abundance, was most strongly linked to mortality, suggesting that qualitative changes in neutrophil composition outweigh quantitative neutrophilia in determining outcome. These findings position immature CD123^+^ neutrophils as a key component of maladaptive alveolar inflammation in ARDS and highlight neutrophil immaturity as a relevant dimension of alveolar immune dysregulation.

Alveolar monocyte/macrophage subset heterogeneity has been shown to associate with many of these different ARDS trajectories. Recent studies leveraging single-cell methods have identified significant heterogeneity in the macrophage subsets present in the immediate period following ARDS, ranging from highly inflammatory recruited monocytes to mature resident macrophages that express more reparative or fibrotic transcriptional programs [24,32,[53], [54], [55], [56], [57], [58], [59], [60]]. Persistence of monocytes and pro-inflammatory macrophages beyond the first week of ARDS have been shown to be associated with worse outcomes [42,49,54]. At the same time, alveolar immune cell exhaustion and impaired phagocytosis have also been associated with poor ARDS outcomes. In a landmark study, Boomer and colleagues demonstrated that immune checkpoint markers such as programmed death-ligand 1 (PD-L1) and programmed death 1 (PD-1) are upregulated on lung antigen presenting cells and T cells, respectively, in patients with sepsis compared with non-septic controls [61]. Human leukocyte antigen-DR (HLA-DR) is responsible for antigen presentation, and its expression on classical monocytes in the blood is significantly depressed in patients with sepsis. Suppressed monocyte HLA-DR expression predicts development of secondary infections and poor outcomes [62,63]. Blockade of other negative co-stimulatory receptors such as cytotoxic T-lymphocyte-associated protein 4 (CTLA-4) in sepsis models has also been shown to decrease sepsis-induced T cell apoptosis and improve outcomes at certain doses [64,65]. Although these findings, and others, have established a clear role for immune paralysis in sepsis, very little is known about the role that immunoparalysis, exhaustion, and tolerances play within the lung during and after ARDS.

### Resolution versus persistent inflammation

The resolution of early “exudative” alveolar inflammation is an active and tightly regulated process, in which efferocytosis represents a key mechanism driving the transition from inflammation to repair. Alveolar macrophages, particularly recruited monocyte-derived macrophages [66], engulf apoptotic neutrophils, which in turn activate numerous downstream transcription factors including liver X receptor alpha (LXR-α), liver X receptor-beta (LXR-β), glucocorticoid-induced leucine zipper protein (GILZ; formerly GC-BP), and peroxisome proliferator-activated receptor-gamma (PPAR-γ), leading to attenuation of proinflammatory cytokine and chemokine expression [[67], [68], [69], [70], [71], [72], [73], [74]]. In parallel, efferocytosis also promotes the release of mediators that interact with the epithelium, such as signal regulatory protein-alpha (SIRP-α) and granulocyte-macrophage colony-stimulating factor (GM-CSF), and promote tissue repair processes, for example via IL-10 and transforming growth factor-beta (TGF-β) [[75], [76], [77], [78]]. Alveolar macrophages are also a key source of pro-resolution lipid mediators such as resolvin D1 and E1, which further attenuate inflammation and promote resolution following the initial exudative phase of ARDS [[79], [80], [81], [82]], although the specific macrophage subsets responsible for producing these mediators are not clear. Disruption of these coordinated processes may contribute to persistent inflammation, impaired repair, and progression towards fibrotic remodeling.

### Repair versus fibrotic remodeling

The later phases of ARDS are highly variable and can range from complete alveolar recovery to profound fibrosis (Fig. 1B.II). In the largest histologic natural history series of patients with ARDS, proliferative changes were defined as “*proliferation of alveolar type 2 (AT2) epithelial cells, interstitial proliferation of fibroblasts and myofibroblasts, or organizing interstitial fibrosis*” [83]. In this study, approximately 70% of lesions during the 2nd week following ARDS onset were categorized as *proliferative* changes. In order for the *proliferative* phase to initiate, there must be active and coordinated resolution of inflammation that is triggered during the proceeding exudative phase. Appropriate differentiation of AT2 into alveolar type 1 (AT1) epithelial cells is a critical step to lung repair following ARDS. The proliferation of AT2 to AT1 cells is regulated by multiple cell types including fibroblasts, endothelial cells, macrophages, and T cells. In animal models of acute lung injury, regulatory T cells (T_reg_) have been shown to play a particularly important role in repair following acute lung injury, in large part through modulation of alveolar cytokines (e.g. interferons (IFNs)), matrix metalloproteinases (MMPs) (e.g. MMP12), growth factors (e.g. TGF-β), and augmenting neutrophil apoptosis [[84], [85], [86]]. T_reg_ depletion has robustly been shown to significantly attenuate AT2 to AT1 differentiation. Alveolar macrophages have also been shown to be a key source of alveolar epithelial growth factors such as hepatocyte growth factor (HGF), keratinocyte growth factor (KGF), and GM-CSF [[87], [88], [89]].

Many of the molecular processes that contribute to post-ARDS alveolar recovery have also been shown to contribute to post-ARDS fibrosis. The factors that lead to repair versus fibrosis are not well defined, but likely involve a combination of host genetic susceptibility, severity of the initial injury (e.g. prolonged mechanical ventilation or hypoxemia), and inability to clear pathogens or the inciting risk factor. For example, prolonged hypoxemia promotes keratin-5 (KRT5)-dependent disorganized repair following influenza infection [90]. Other mediators such as keratin-8 (KRT8) and Wnt signaling that play a key evolutionary role in tissue repair have been implicated in lung fibrosis when excessively or inappropriate activated [91,92].

## Complications of alveolar immune dysfunction: secondary infection and reactivation

### Bacterial ventilator-associated pneumonia

Ventilator-associated pneumonia is characterized by a neutrophil-dominated inflammatory response within the alveolar compartment (Fig. 1C.I). Studies using bronchoalveolar lavage have shown that local cytokine patterns can discriminate infection more accurately than systemic biomarkers. Elevated concentrations of IL-1β and IL-8 in BAL fluid are strongly associated with microbiologically confirmed VAP, while their absence effectively rules out infection [27,93]. These findings underline that although systemic involvement may be present, peripheral blood biomarkers do not adequately reflect the presence and magnitude of alveolar infection. They also highlight that diagnostic approaches focusing on compartment-specific cytokine responses can provide a more accurate assessment of lower respiratory tract infection than systemic parameter.

Alongside cytokine activation, alveolar neutrophils in VAP display strong effector activity with marked release of proteases and matrix-degrading enzymes such as elastase and metalloproteinases, although phagocytosis is often impaired [11]. This reflects an effective but potentially injurious attempt to eliminate pathogens in a closed environment. Local tumor necrosis factor (TNF)-axis activation, evidenced by increased soluble TNF-receptor expression, further indicates cross-talk between epithelial and myeloid cells that amplifies both pathogen clearance and tissue injury [94]. Together, these observations suggest that the host response in VAP involves a tightly regulated but easily imbalanced network of inflammatory mediators that can drive epithelial barrier damage and perpetuate infection even after initial microbial control.

Importantly, alveolar immune dysfunction in VAP does not arise de novo at the time of infection. Impaired neutrophil function, reduced complement receptor expression, and defective phagocytosis have been observed in critically ill patients before the onset of VAP and are associated with subsequent infection risk [11,95]. These findings suggest that systemic inflammation and critical illness–induced immune alterations create a permissive alveolar environment in which pathogens can persist and flourish. Recent longitudinal analyses combining microbiome and host-response data indicate that both microbial composition and inflammatory programs evolve dynamically during mechanical ventilation, preceding overt infection [96]. Together, these studies support a model in which VAP results from a progressive breakdown of local immune balance rather than a single infectious insult, emphasizing the need for serial, compartment-focused monitoring to detect early immune failure and guide timely intervention.

### Viral reactivation

The disrupted alveolar immune environment in patients with lung injury creates an opportunity for viral and fungal pathogens to thrive. Prolonged immune dysregulation facilitates reactivation of latent viruses and the emergence of secondary infections, a phenomenon long recognized in immunocompromised patients but also observed in critically ill patients without overt immunosuppression [97,98]. These pathogens not only benefit from lung injury, but can also sustain or further aggravate it, amplifying inflammation and reinforcing a self-perpetuating cycle of lung tissue damage.

Viral reactivations in the lung compartment, in particular herpes simplex virus (HSV) and cytomegalovirus (CMV), are commonly observed in patients with prolonged ICU stays [[97], [98], [99], [100]]. Their occurrence is not limited to classical immunocompromised states, but is also observed in critically ill patients with ARDS, sepsis, and those receiving prolonged invasive mechanical ventilation and corticosteroids [99,[101], [102], [103], [104]]. Pulmonary herpes virus reactivations were associated with worse clinical trajectories in observational cohorts: longer ICU stays, prolonged duration of mechanical ventilation, fewer ventilator-free days [[103], [104], [105], [106], [107], [108], [109], [110], [111]]. They have also been linked to an increased risk of secondary infections, including VAP and COVID-19 associated pulmonary aspergillosis (CAPA) [100,104,105,112,113]. These complications underline that pulmonary viral reactivation is at minimum a marker of impaired alveolar immunity and overall patient vulnerability.

A solid association with mortality is difficult to establish, as inconsistent findings in literature largely reflect methodological heterogeneity, use of non-quantitative diagnostics, and inadequate adjustment for time-dependent biases. In addition, the absence of standardized viral load thresholds contributes to variability in reported incidence and outcomes and limits clinical interpretation. Two studies that addressed these methodological issues, in COVID-19 ARDS and all-cause VAP cohorts, reported an association between pulmonary HSV reactivation and mortality based on quantitative viral loads and time-dependent models [105,106]. By contrast, none of the well-designed studies have shown an association of CMV reactivation in the alveolar compartment with mortality, with described associations limited to blood or a retracted study [111,114]. Although antiviral treatment seems to be associated with a reduced viral load (and increased VFDs) [115], none of the randomized trials of antivirals published to date have found a mortality benefit in treating Herpesviridae reactivations [116]. However, this does not preclude the possibility of subgroups that may benefit or smaller-than-anticipated effect sizes. Ongoing trials, including the multicenter HerpMV study, are currently evaluating whether targeted antiviral therapy improves clinical outcomes in patients with a pulmonary HSV reactivation [117].

Effective suppression of HSV and CMV latency relies on a coordinated antiviral immune response, involving interferon signaling, cytotoxic T cells, and alveolar macrophages [118,119]. These elements normally act together to prevent viral replication while preserving tissue integrity. In patients with acute lung injury, however, this delicate balance is disrupted: hyperinflammation and subsequent immune exhaustion blunt interferon pathways, weaken T cell responses [39,120], and drive alveolar macrophages toward a dysregulated, proinflammatory state [37,121]. This immune dysregulation provides a permissive environment for both HSV and CMV reactivation. (Fig. 1C.II)

Yet, important differences exist. HSV, latent in peripheral neurons, combines loss of immune control with rapid replication and additional triggers such as mucosal injury from intubation and ventilation, leading to early and frequent pulmonary reactivation in ARDS patients [122]. Following pulmonary HSV-1 reactivation, an increase in activated CD8 + T cells has been observed, alongside a loss of interferon-driven control [123]. This paradox reflects a hyperactivated but ineffective immune response that permits viral replication and epithelial injury.

CMV, in contrast, establishes latency in myeloid progenitors and endothelial cells and has a slower replication cycle, with reactivation predominantly linked to T cell exhaustion. This tends to become clinically relevant later in the disease course and may therefore be more important in settings of chronic immunosuppression, such as transplant recipients, than in the typical ARDS ICU setting [124].

Together, these observations raise the question whether adverse outcomes in ARDS are primarily the consequence of immune failure, viral replication itself, or a combination of both—an issue that remains unresolved. This is partly because studies addressing herpesvirus pathophysiology in the alveolar compartment are still scarce.

### Fungal infections

Fungal coinfections, particularly pulmonary aspergillosis, represent another complication arising from alveolar immune dysregulation in ARDS. While pulmonary aspergillosis classically occurs in immunocompromised hosts, it is increasingly recognized in immunocompetent ICU patients with severe viral pneumonia, particularly influenza and COVID-19, where it is referred to as virus-associated pulmonary aspergillosis (VAPA). This term encompasses both influenza-associated (IAPA) and COVID-19–associated pulmonary aspergillosis (CAPA). In rigorously sampled cohorts, VAPA is diagnosed in up to 20% of patients with influenza or COVID-19 ARDS [125]. Compared to viral ARDS patients without fungal coinfection, VAPA is associated with a twofold increase in mortality, with an overall mortality rate of approximately 50% [126,127]. To a lesser extent, pulmonary aspergillosis has been observed to complicate or cause ARDS in other ICU patients lacking classical risk factors, such as those with pneumonia due to bacterial or other viral pathogens [128,129], burns [130], or cirrhosis [131].

How alveolar dysregulation in ARDS predisposes to fungal coinfections has been best studied in the context of influenza and COVID-19 ARDS. Viral replication induces epithelial injury and a hyperinflammatory milieu that facilitates Aspergillus growth. At the same time, a dysregulated alveolar response to viral replication compromises multiple layers of antifungal defense. Epithelial barrier disruption permits hyphal invasion, while monocytes and macrophages exhibit impaired activation, with reduced phagocytosis, reduced reactive oxygen species generation, and cytokine release, thereby allowing inhaled conidia to germinate and form hyphae [[132], [133], [134]] (Fig. 1C.III).

The normal alveolar response to aspergillus hyphae is hallmarked by extensive neutrophilic inflammation [135]. Despite neutrophilic infiltration, the neutrophilic response to Aspergillus in viral ARDS might be exhausted, with downregulation of neutrophil effector pathways and dysfunctional NET formation [132,136]. The latter is further supported by the observation that increased levels of citrullinated histone H3 DNA, a marker of effective NET release, correlated with improved survival in VAPA patients and likely represents more effective NET formation [136].

At the level of the adaptive immune system, influenza and COVID-19 reduce trajectory maturation of Th1 and Th17 cells, which normally support phagocyte activation and epithelial repair, while mucosal-associated invariant T cells, important for early antifungal responses, are depleted [136]. Finally, therapeutic interventions aimed at controlling inflammation in ARDS, such as corticosteroids, may iatrogenically weaken the antifungal immunity and increase susceptibility to IPA [137].

Together, these defects converge to create a permissive alveolar niche in which Aspergillus can germinate and proliferate, amplifying tissue injury and perpetuating a cycle of dysregulated inflammation and secondary infection.

## Implications

### Sampling and biomarker assessment

The recognition that alveolar and systemic host responses are frequently discordant has important implications for both research and clinical care. While bronchoalveolar lavage provides direct access to the alveolar compartment, its invasive nature limits routine and longitudinal use in critically ill patients. As a result, there is growing interest in noninvasive or minimally invasive approaches, such as endotracheal aspirates and molecular profiling techniques, to approximate lower respiratory tract biology.

A recent American Thoracic Society workshop report on respiratory sampling in acute respiratory failure highlights key research priorities, including the need for standardization of sampling and processing protocols, validation against alveolar reference samples, and rigorous head-to-head comparisons between sampling methods [138]. Although noninvasive approaches may improve feasibility and scalability, important uncertainties remain regarding their anatomical specificity, reproducibility, and the extent to which they reflect true alveolar processes. At present, selection of sampling methods is largely driven by practical considerations rather than validated biological criteria. This further complicates the interpretation of findings in conditions such as fungal infections and viral reactivation, where distinguishing colonization, reactivation, and invasive disease remains challenging.

Systemic biomarker-based approaches may not fully capture lung-specific immune processes. Circulating inflammatory signals may decline over time, while alveolar inflammation persists, particularly in later phases of critical illness. This temporal and spatial dissociation may lead to underestimation of clinically relevant alveolar inflammation when relying on blood-based measurements alone.

Bridging invasive and noninvasive sampling strategies will be essential to enable longitudinal assessment of lung-specific host responses and to facilitate their integration into clinical studies. Such approaches may support prognostic and predictive enrichment, improve patient stratification, and guide the development of targeted immunomodulatory and pathogen-directed therapies. This is particularly relevant given the dynamic and compartment-specific nature of alveolar immune responses described above.

### Clinical decision-making and personalized therapy

These insights have direct implications for clinical decision-making. Systemic and alveolar immune responses may reflect different domains of disease severity. While systemic inflammation has been associated with extra-pulmonary organ dysfunction and early mortality, the relationship between alveolar inflammation and clinical outcomes remains less well defined, but may relate more closely to lung-specific outcomes such as prolonged respiratory failure. Current approaches to precision medicine in critical illness are largely being developed and evaluated within the research setting, with a strong focus on systemic biomarker-based stratification, for example distinguishing hyper- and hypoinflammatory phenotypes to guide immunomodulatory therapies (such as corticosteroids). Emerging bedside biomarker platforms may support future clinical implementation of these strategies. Previous studies have demonstrated improved survival with corticosteroid therapy in patients with a hyperinflammatory systemic subphenotype [113,139,140]. In the context of compartment-specific immune responses, this raises the possibility that patients with a hyperinflammatory alveolar phenotype - regardless of systemic classification - may represent a population that could potentially benefit from lung-targeted immunomodulatory treatment.

**Fig. 2:**
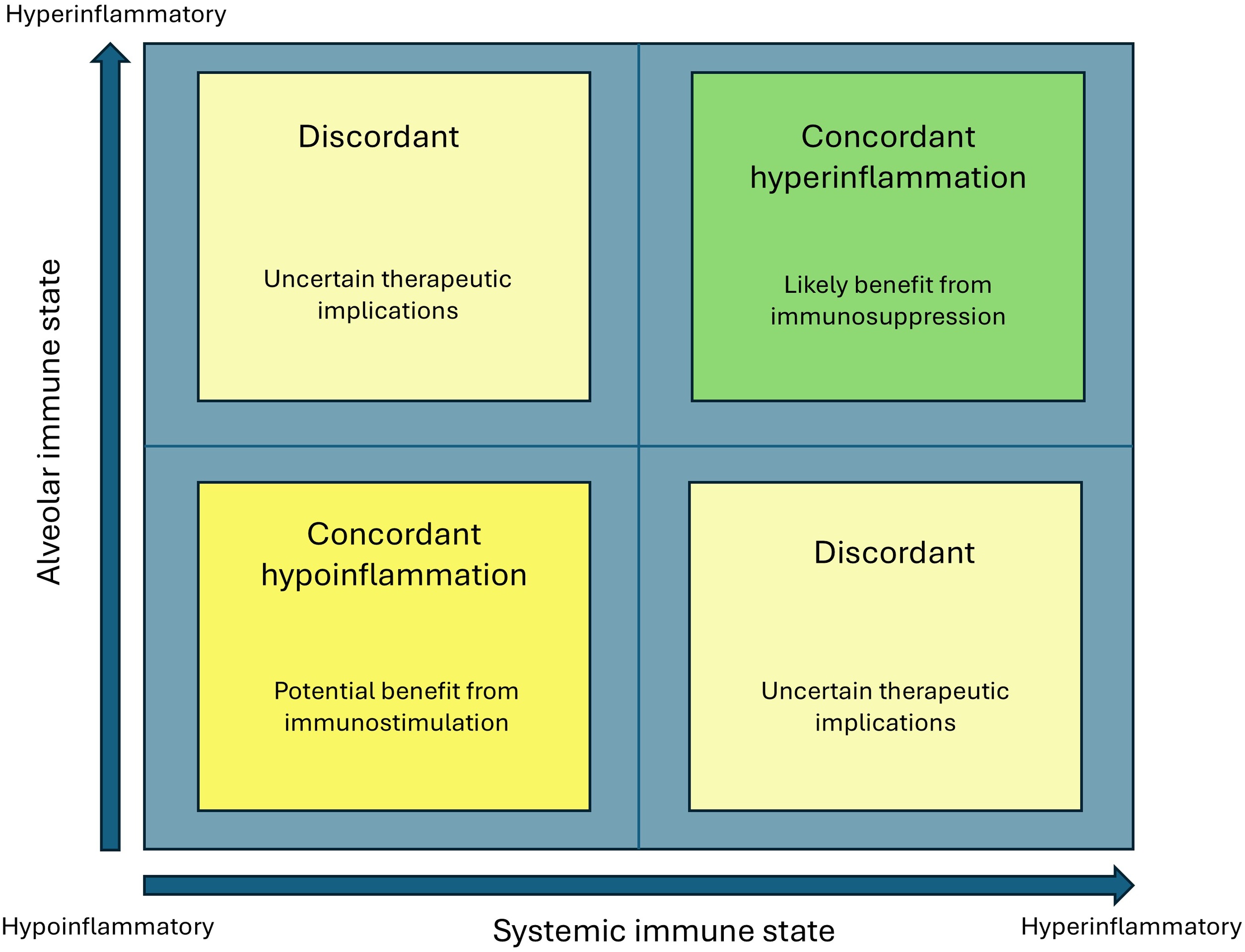
Conceptual framework of compartment concordance and discordance. Concordance between systemic and alveolar immune states may guide therapy, whereas discordance reflects uncertainty regarding optimal treatment strategies. The x-axis represents the systemic immune state, ranging from hypoinflammatory (left) to hyperinflammatory (right), and the y-axis represents the alveolar immune state, ranging from hypoinflammatory (bottom) to hyperinflammatory (top).

However, discordance between systemic and alveolar immune responses may limit the utility of such strategies. Systemic phenotyping may fail to identify patients with persistent or clinically relevant alveolar inflammation, potentially leading to undertreatment of injurious lung inflammation, while in other cases anti-inflammatory therapy may be applied despite relative pulmonary immune suppression. These observations support a more personalized and compartment-informed approach, in which both systemic and alveolar immune states (and their concordance) are considered when selecting treatment strategies [141] (Fig. 2). In this context, current precision-medicine efforts, including biomarker-guided platform trials, illustrate the feasibility of phenotype-directed treatment strategies, but prospective studies incorporating alveolar phenotyping are needed to determine whether this approach improves treatment selection.

### Treatable traits and future studies

In addition to immune phenotyping, the role of secondary infections highlighted in this review underscores the importance of identifying treatable traits within the alveolar compartment. Secondary infections, including bacterial ventilator-associated pneumonia, viral reactivation, and fungal co-infection, may arise in the context of impaired local host defense and represent actionable therapeutic targets. Targeted diagnostics to identify such processes may allow for more precise interventions, such as antiviral or antifungal therapy, or refinement of immunomodulatory strategies based on underlying alveolar processes. Ongoing randomized controlled trials are currently evaluating targeted treatment strategies for viral reactivation, reflecting increasing recognition of these processes as potential therapeutic targets [117].

Future studies should therefore aim to validate alveolar subphenotyping in broader ARDS populations, assess concordance and discordance between systemic and alveolar immune states longitudinally, and develop feasible bedside biomarker platforms for compartment-specific phenotyping. These efforts could support interventional trials that target patients based on combined systemic and alveolar profiles, rather than systemic biomarkers alone, and thereby move the field closer to a more dynamic and personalized approach to ARDS management. Together, these considerations are summarized in Table 2, outlining key clinical implications and future research directions.

**Table 2:** Summary of key clinical implications and potential future trials.

Domain	Key implication	Potential clinical application	Future study/trial direction
Compartment discordance	Blood may not reflect alveolar immune state	Avoid treatment decisions based on systemic biomarkers alone	Prospective studies comparing blood vs alveolar phenotyping
Alveolar subphenotyping	Hyper-/hypoinflammatory profiles may differ by compartment	More precise patient stratification for immunomodulation	Validation of alveolar phenotypes in multicenter cohorts
Bedside biomarker testing	Rapid phenotyping may enable real-time treatment selection	Use platforms such as point-of-care biomarker panels	Trials integrating bedside systemic and alveolar phenotyping
Treatable traits	Secondary infections may drive non-resolving injury	Viral/fungal diagnostics; targeted antiviral/antifungal treatment	Trials on HSV/CMV/aspergillosis-guided treatment in ICU setting
			

Abbreviations: HSV, herpes simplex virus; CMV, cytomegalovirus; ICU, intensive care unit.

## Conclusions

The alveolar compartment is a key determinant of outcome in pneumonia and ARDS, functioning as an active immunological niche in which epithelial injury and innate immune responses interact in self-sustaining cycles that may resolve or perpetuate lung injury. Across these syndromes, local immune responses are frequently discordant from systemic signatures and are not adequately captured by peripheral biomarkers alone.

These alveolar processes evolve along heterogeneous and dynamic trajectories, from resolution and epithelial repair to persistent hyperinflammation, immune suppression, or fibrotic remodeling. Ineffective alveolar host defense manifests clinically as persistent lung injury and secondary infections, including bacterial ventilator-associated pneumonia, viral reactivation, and fungal co-infection, reflecting a shared dysregulation of local immunity.

Recognition of compartment-specific immune responses provides a framework to better understand clinical heterogeneity and to identify treatable traits within the lung. Integrating systemic and alveolar immune profiling may ultimately enable more personalized, compartment-informed approaches to diagnosis, risk stratification, and targeted therapy.

## Authors' contributions

LDJB was invited as corresponding principal investigator to lead the development of this review. He defined the scope and conceptual framework of the manuscript, selected and coordinated the contributing authors, and supervised the integration of all sections into a unified narrative. LSB coordinated the manuscript structure, synthesized the individual sections, and harmonized the overall text. LSB, LM, JW, EM, AS, ACM, and LDJB each drafted the section aligned with their respective area of expertise. LM and LSB also designed the figures. All authors critically revised the manuscript for important intellectual content and approved the final version.

## Consent for publication

Not applicable.

## Ethics approval and consent to participate

Not applicable; this manuscript is a narrative review based exclusively on previously published studies and did not involve the collection of new data from human participants or animals.

## Declaration of Generative AI and AI-assisted technologies in the writing process

Generative AI (ChatGPT-5) was used only for limited language editing of final drafts. All scientific content and interpretation were produced by the authors without AI assistance.

## Funding

ACM is supported by an MRC Clinician Scientist Fellowship (MR/V006118/1). EDM is supported by 10.13039/100000050NHLBIR01 HL169265. LM received funding from Research Foundation Flanders (grant number 11A0K26N). LDJB is supported by the NWO VIDIgrant “Navigating treatment response in acute respiratory distress syndrome using a biological compass” (grant number: 09150172210004).

## Availability of data and material

Not applicable; this manuscript is a narrative review based on previously published literature.

## Declaration of competing interest

ACM sits on the scientific advisory board of Cambridge Infection Diagnostics and has received speaking fees from Thermo-Fisher, Biomerieux, Fischer and Paykel, and Boston Scientific and participated on advisory boards for Mundipharma and Abbott Diagnostics. All fees are paid to ACM’s institution. All remaining authors report no conflicts of interest.
